# Small-molecule PIK-93 modulates the tumor microenvironment to improve immune checkpoint blockade response

**DOI:** 10.1126/sciadv.ade9944

**Published:** 2023-04-07

**Authors:** Chia-Yi Lin, Kuo-Yen Huang, Shih-Han Kao, Ming-Shiu Lin, Chih-Chien Lin, Shuenn-Chen Yang, Wei-Chia Chung, Ya-Hsuan Chang, Rong-Jie Chein, Pan-Chyr Yang

**Affiliations:** ^1^ Department of Internal Medicine, College of Medicine, National Taiwan University, Taipei 100, Taiwan.; ^2^ Institute of Biomedical Sciences, Academia Sinica, Nankang, Taipei 115, Taiwan.; ^3^ Department of Clinical Laboratory Sciences and Medical Biotechnology, College of Medicine, National Taiwan University, Taipei, Taiwan.; ^4^ Resuscitation Science Center of Emphasis, Department of Anesthesiology and Critical Care Medicine, The Children’s Hospital of Philadelphia, Philadelphia, PA 19104, USA.; ^5^ Institute of Statistical Science, Academia Sinica, Taipei, Taiwan.; ^6^ Institute of Chemistry, Academia Sinica, Nankang, Taipei 115, Taiwan.; ^7^ Genomics Research Center, Academia Sinica, Nankang, Taipei 115, Taiwan.

## Abstract

Immune checkpoint inhibitors (ICIs) targeting PD-L1 immunotherapy are state-of-the-art treatments for advanced non–small cell lung cancer (NSCLC). However, the treatment response of certain patients with NSCLC is unsatisfactory because of an unfavorable tumor microenvironment (TME) and poor permeability of antibody-based ICIs. In this study, we aimed to discover small-molecule drugs that can modulate the TME to enhance ICI treatment efficacy in NSCLC in vitro and in vivo. We identified a PD-L1 protein-modulating small molecule, PIK-93, using a cell-based global protein stability (GPS) screening system. PIK-93 mediated PD-L1 ubiquitination by enhancing the PD-L1–Cullin-4A interaction. PIK-93 reduced PD-L1 levels on M1 macrophages and enhanced M1 antitumor cytotoxicity. Combined PIK-93 and anti–PD-L1 antibody treatment enhanced T cell activation, inhibited tumor growth, and increased tumor-infiltrating lymphocyte (TIL) recruitment in syngeneic and human peripheral blood mononuclear cell (PBMC) line–derived xenograft mouse models. PIK-93 facilitates a treatment-favorable TME when combined with anti–PD-L1 antibodies, thereby enhancing PD-1/PD-L1 blockade cancer immunotherapy.

## Teaser

Small-molecule PIK-93 modulates PD-L1 stability though CUL4A and benefits the tumor microenvironment for immunotherapy.

## INTRODUCTION

Lung cancer is the leading cause of cancer-related death worldwide (*1*). Although targeted therapy against driver mutations has successfully improved the disease outcomes in patients with non–small cell lung cancer (NSCLC), the majority of patients without actionable driver mutations rely on traditional chemotherapies. Nevertheless, the response rate to traditional chemotherapy is far from satisfactory. Recent advances have shown that tumor growth requires interactions among tumor cells, the surrounding tumor microenvironment (TME), host immunity, etc. Blocking the cross-talk between tumor and immune cells can thus diminish aggressive tumor growth. Therapies such as immune checkpoint inhibitors (ICIs) block inhibitory signals sent from tumor cells to tumor-infiltrating lymphocytes (TILs), allowing the activation of T cells that kill cancer cells. The best characterized checkpoint proteins expressed on T cells and tumor cells, respectively, are Programmed Death-1 (PD-1)/Programmed Death-ligand 1 (PD-L1). Immunotherapy targeting PD-1/PD-L1 has become the state-of-the-art, efficacious treatment for advanced NSCLC (*2*). However, still many patients show suboptimal responses to PD-1/PD-L1 therapy. Therefore, it is pivotal to develop combination therapies to enhance the efficacy of ICI treatment.

One of the reasons why PD-1/PD-L1 therapy has a limited response rate is due to the chemical nature of the inhibitors. PD-1/PD-L1 inhibitors are antibody-based drugs that exhibit inadequate pharmacokinetics and poor permeability in tumor tissues. Other drawbacks of these antibody-based drugs include unwanted immunogenicity against antibodies, lack of oral bioavailability, and costly, time-consuming production (*3*). Therefore, the limitations of antibody drugs have led to the development of PD-1/PD-L1–based small-molecule inhibitors. Notably, small-molecule–induced targeted protein degradation is a powerful therapeutic approach, especially for undruggable proteins through conventional therapeutic strategies (*4*). Several anticancer small molecules have shown clinical efficacy by mediating protein degradation; these agents include lenalidomide (*5*) and thalidomide (*6*), with previously unidentified discoveries continually entering the clinic (*7*).

The ubiquitin (Ub)–proteasome pathway is a major regulatory pathway of protein metabolism. The addition of Ub to a target protein, a process called ubiquitination, is mediated by the sequential action of the E1 Ub-activating enzyme, E2 Ub-conjugating enzyme, and E3 Ub ligase (*8*). E3 ligases play a crucial role in determining the substrate specificity in the Ub-proteasome system (*8*). The largest family of E3 ligases is Cullin-RING ligases (CRLs), responsible for the ubiquitination and proteasome degradation of ~20% of cellular proteins (*9*). Among these ligase functions, CRL4s promote viral infections and DNA metabolism (*10*), but the roles of CRL4s in antitumor immunity remain unclear.

Various immune cell types participate in cancer immune evasion; these cells include regulatory T cells (*11*), tumor-associated macrophages (TAMs) (*12*), and myeloid-derived suppressor cells (*13*) in the TME. Among these cells, TAMs play an essential role in tumor progression (*14*), chemotherapy resistance, and immunosuppression (*15*). TAMs expressing the ligands of PD-1 and cytotoxic T-lymphocyte–associated protein 4 (CTLA4), including PD-L1, PD-L2, B7H4, B7-1, and B7-2, contribute to the immunosuppressive functions of macrophages (*16*). TAMs can also regulate PD-L1 expression on tumors and PD-L1–related cytokine expressions in the TME (*17*). Notably, macrophages can be polarized into the classically activated M1-type or alternatively activated M2-type macrophages, as mediated by specific microenvironmental signals and stimuli (*18*). M1 TAMs are associated with tumor cell killing and proinflammatory cytokine production (*19*), whereas M2 TAMs secrete anti-inflammatory cytokines and promote angiogenesis and tumor growth (*20*). Recently, a high M1/M2 ratio in patients has been reported to be associated with improved survival (*21*, *22*), implicating that M1 TAMs may aid the effect of cancer treatment.

Here, we integrated a high-throughput global protein stability (GPS) and identified a candidate drug, PIK-93, that can degrade PD-L1 proteins. The potential mechanism suggests that PIK-93 enhances the interaction of Cullin-4A (CUL4A) with PD-L1 and induces ubiquitination and proteasome-mediated degradation of PD-L1. We further demonstrated that PIK-93–treated polarized THP-1–derived M1 macrophages showed reduced PD-L1 levels and enhanced cytotoxicity, significantly expanding the repertoire of known cells that PIK-93 modulates to achieve immune checkpoint blockade. We characterized the in vivo effects of PIK-93 on tumor suppression and T cell recruitment in the TME in syngeneic and human peripheral blood mononuclear cell (PBMC) line–derived xenograft (CDX) mouse models.

## RESULTS

### A cell-based global protein stability screening system is established to screen compounds that can modulate PD-L1 expression

**Fig. 1.**
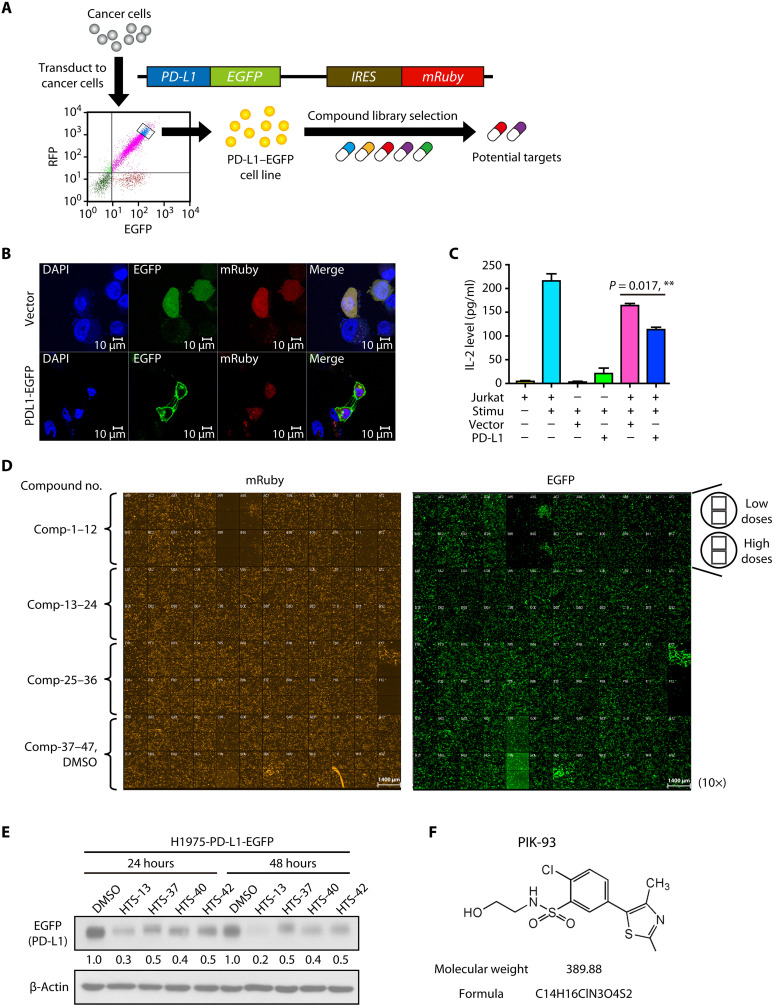
HTS of drugs combined with the PD-L1–EGFP cell system to select unique PD-L1 degradation compounds. (**A**) The concept underpinning the PD-L1–enhanced green fluorescent protein (EGFP) cell selection system. H1975 cells stably expressing PD-L1–EGFP were sorted by flow cytometry. RFP, red fluorescent protein; IRES, internal ribosome entry site. (**B**) Immunofluorescence staining of stable H1975–PD-L1–EGFP–expressing and H1975-vector–expressing cells. DAPI, 4′,6-diamidino-2-phenylindole. (**C**) The interleukin-2 (IL-2) production assay of Jurkat T cells with or without coculture with H1975–PD-L1–EGFP–expressing cells. H1975–PD-L1–EGFP–expressing and H1975–vector–expressing stable cells were cocultured with activated Jurkat T cells with or without the Cell Stimulation Cocktail for 24 hours. The levels of IL-2 were measured by enzyme-linked immunosorbent assay (ELISA). The data are shown as the means and SEM for technical and biological three independent experiments (*n* = 3). Significant differences between indicated groups were calculated by Student’s *t* test (**P < 0.01). (**D**) Fluorescence images taken 24 hours after high-throughput screening (HTS) of 47 different compounds in H1975–PD-L1–EGFP cells. (**E**) Cells stably expressing PD-L1–EGFP were treated with HTS-13, HTS-37, HTS-40, and HTS-42 candidate compounds for 24 or 48 hours. The cells were harvested, and EGFP expression levels were evaluated by immunoblotting. β-Actin was used as an internal control. DMSO, dimethyl sulfoxide. (**F**) Structure of PIK-93.

We established a drug-screening platform using a cell-based fluorescent reporter system called the GPS platform (*23*). The fluorescent-based retroviral reporter system can be used to examine the changes in protein stability systematically. The GPS vectors express a single transcript that encodes an enhanced green fluorescent protein (EGFP) fusion protein and mRuby separated by an internal ribosome entry site (IRES). The EGFP/mRuby ratio in each cell is an indicator of the half-life of the expressed open reading frame (i.e., *PD-L1* in this study). Following PD-L1–EGFP-mRuby plasmid transduction, H1975 cells were sorted three times based on the EGFP/mRuby ratio by flow cytometry (Fig. 1A). Our data showed that the expressed PD-L1–EGFP proteins were mainly localized on the cell membrane (Fig. 1B) and could functionally inhibit interleukin-2 (IL-2) production by coculture with human leukemia cells, Jurkat T cells (Fig. 1C). IL-2 secretion by Jurkat T cells is a key indicator of ICI immunotherapy effects. The established cell system serves as the screening platform for compound drugs, as assessed by changes in fluorescence intensity, which implicates changes in the stability of the proteins of interest.

### Candidate PD-L1–modulating drugs are identified by high-content imaging-based screening using the PD-L1–EGFP cell system

To screen candidate drugs that can modulate PD-L1 expression, we observed the fluorescence intensity of H1975–PD-L1–EGFP in cells with a high-content imaging-based screening system using a compound library established in our laboratory (Fig. 1D). The half-maximal inhibitory concentration (IC_50_) values were calculated on the basis of EGFP/red fluorescent protein (RFP) fluorescence intensity (Supplementary Materials and fig. S1). Compounds at low dose with <50% survival and without dose response were excluded. To confirm high-throughput screening (HTS) findings, we examined EGFP (PD-L1) expression by Western blotting and found that the selected candidates, HTS-13, HTS-37, HTS-40, and HTS-42, decreased EGFP (PD-L1) expression (Fig. 1E). However, previous studies have shown that HTS-13 and HTS-40 are mammalian target of rapamycin (mTOR) inhibitors, which induce apoptosis in hematopoietic lineage cells (*24*, *25*), and that HTS-42 suppresses T cell activation (*26*). Our study goal is to discover a unique compound that can modulate the TME by inducing PD-L1 degradation to improve immune checkpoint blockade response. Therefore, candidates with a high cytotoxic effect or a side effect on T cells were excluded from our study. We identified HTS-37 as PIK-93, which is a phenyl-based small molecule (Fig. 1F), and it has been reported to inhibit the activity of phosphatidylinositol 3-kinases (PI3Ks), including PI3Kα, PI3Kβ, PI3Kγ, and PI3Kδ (*27*) and to inhibit phosphatidylinositol 4-kinase IIIβ (PI4Kβ) selectively. Therefore, we further explored the mechanism of action of PIK-93 on PD-L1.

### PIK-93 reduces PD-L1 expression and induces low cytotoxicity in NSCLC cells

**Fig. 2.**
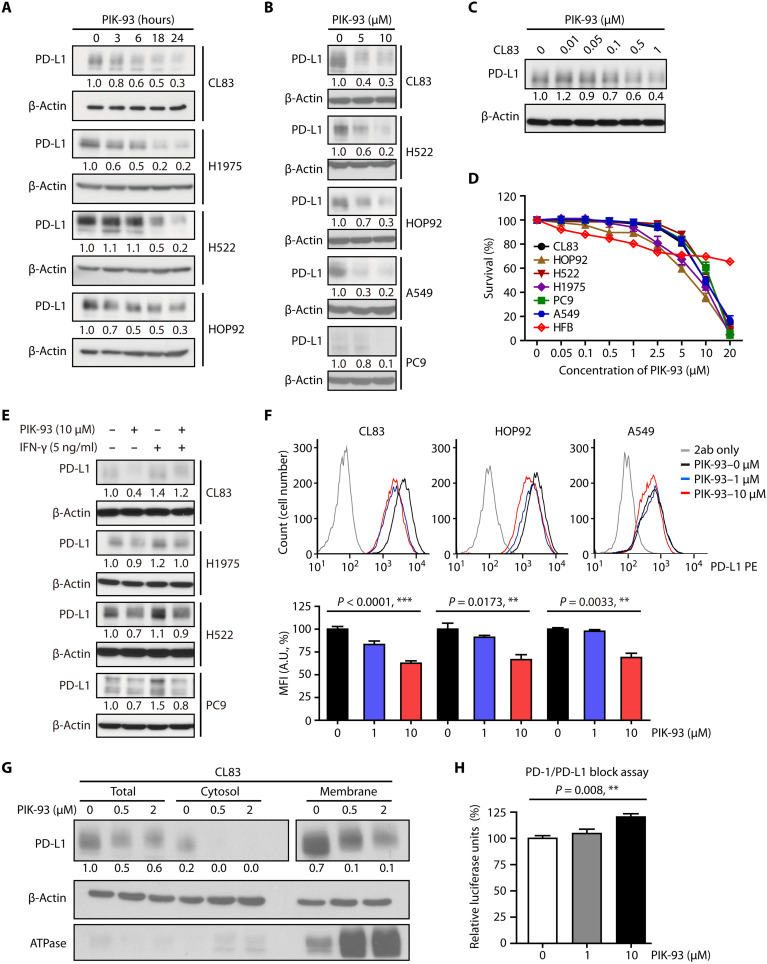
PIK-93 induces membrane-bound and cytosolic PD-L1 degradation to activate T cells. (**A**) Increased degradation of PD-L1 in CL83, H522, HOP92, and H1975 cells after 1 μM PIK-93 treatment. Cells were harvested at the indicated time points, and the levels of PD-L1 were analyzed by immunoblotting. (**B**) Dose-dependent degradation of PD-L1 in different lung cancer cell lines. Cells were treated with the indicated doses of PIK-93 for 24 hours and then harvested. The PD-L1 level was determined by immunoblotting. (**C**) CL83 cells were treated with the indicated concentrations of PIK-93 for 24 hours before harvest, and the PD-L1 level was analyzed by immunoblotting. (**D**) The cytotoxicity assay after PIK-93 treatment in various cell lines. Cells were treated with 0 to 20 μM PIK-93 for 72 hours, and survival was assessed by the sulforhodamine B (SRB) assay. (**E**) PIK-93 decreases interferon-γ (IFN-γ)–induced PD-L1 expression. CL83, H1975, H522, and PC9 cells were treated with IFN-γ (0 or 5 ng/ml) with or without PIK-93 for 24 hours. (**F**) CL83, HOP92, and A549 cells were treated with 0, 1, and 10 μM PIK-93 for 24 hours, and membrane-bound PD-L1 (mPD-L1) was detected by flow cytometry. MFI, mean fluorescent intensity; A.U., absorbance units. (**G**) CL83 cells were treated with 0, 0.5, and 2 μM PIK-93 for 24 hours before being harvested for membrane protein extraction. ATPase, adenosine triphosphatase. (**H**) PIK-93 treatment decreases the PD-1/PD-L1 interaction and restores T cell activity. CHO-K1 cells overexpressing PD-L1 were pretreated with 0, 1, and 10 μM PIK-93 for 24 hours and subsequently cocultured with Jurkat T cells for another 6 hours. The relative luciferase signal indicates Jurkat T cell activity. The results in (D), (F) and (H) are expressed as means ± SEM for biological three independent experiments (*n* = 3). Significant differences between indicated groups were calculated by Student’s *t* test (**P < 0.01 and ***P < 0.001).

We next determined whether PIK-93 treatment modulates the expression of PD-L1 in different NSCLC cell lines. The results indicated that PIK-93 gradually reduced PD-L1 levels in lung adenocarcinoma cell lines, including H1975, CL83, H522, and HOP92 cell lines (Fig. 2A). PIK-93 also dose-dependently reduced PD-L1 (Fig. 2B), and the minimal dosage of PIK-93 that decreased PD-L1 expression was found to be 0.1 μM (Fig. 2C). However, PIK-93 did not affect the RNA level of PD-L1 in the above-mentioned cell lines (fig. S2), suggesting that PD-L1 may undergo protein degradation by PIK-93. To further confirm that PIK-93–induced PD-L1 degradation is not associated with the PI3K pathway, we treated CL83 cells with a PI3K inhibitor, LY294002, for 24 hours. The result showed that only PIK-93 reduced PD-L1 levels (fig. S3, A and B).

To assess whether treatment with PIK-93 modulates other immune checkpoint molecule expressions, we examined CTLA4 and T-cell immunoglobulin and mucin domain 3 (TIM3) protein expressions by Western blotting. CTLA4 and TIM3 expressions did not change after PIK-93 treatment for 24 hours in CL83, H1975, and H522 cells. Moreover, the expression of CD86, the corresponding immune ligand of CTLA4, was not reduced, but the expression of CERCAM-1, the ligand of TIM3, was slightly reduced in CL83 and H522 cancer cell lines (fig. S3, C and D). Together, our finding suggests that PD-L1 is the major immune checkpoint molecule that is modulated by PIK-93.

To explore whether PIK-93–mediated reduction of PD-L1 protein expression results from cytotoxicity, we investigated PIK-93–mediated cell survival in CL83, HOP92, H522, H1975, PC9, and A549 lung cancer cell lines and the HFB normal fibroblast cell line by the sulforhodamine B (SRB) assay. PIK-93 was found to have little cytotoxicity in lung cancer cells with IC_50_ values of >5 μM and in normal fibroblast cells with IC_50_ values of >20 μM (Fig. 2D). Together, these data suggest that PIK-93 reduces PD-L1 expression while exerting little cytotoxic effects on NSCLC cells.

### PIK-93 decreases IFN-γ–induced PD-L1 through proteasome degradation

Interferon-γ (IFN-γ) has been reported to be a cytokine secreted from inflammatory cells in the TME and a key modulator of PD-L1 expression in various types of cell lines (*28*). To verify that PIK-93 can modulate IFN-γ–induced PD-L1 expression, we cotreated CL83, H522, H1975, and PC9 cells with IFN-γ in the presence or absence of PIK-93 for 24 hours. As shown in Fig. 2E, PD-L1 expression was up-regulated by IFN-γ treatment and decreased by PIK-93 cotreatment. These results indicate that PIK-93 can regulate PD-L1 expression mediated by IFN-γ.

### PIK-93 inhibits cytosolic and membrane-bound PD-L1 expressions and restores T cell activity

To assess whether treatment with PIK-93 modulates PD-L1 expression on the cell surface, we first examined PD-L1 membrane expression by flow cytometry. The membrane-bound PD-L1 (mPD-L1) expressions after PIK-93 treatment for 24 hours were significantly reduced in CL83, HOP92, and A549 cells (Fig. 2F). We next performed cell fractionation, and in accordance, membrane-bound and cytosolic PD-L1 expression levels were both reduced by PIK-93 in subcellular fractions of CL83 cells (Fig. 2G).

In the TME, PD-L1 on tumor cells binds to PD-1 on T cells, leading to the inhibition of cytotoxic T cell activity. To investigate whether the reduced expression of PD-L1 would lead to recovered T cell activity, we used the PD-1/PD-L1 blockade bioassay kit that measures the effectiveness of compounds designed to block the PD-1/PD-L1 interaction (*29*) by coculture the Jurkat T cells offering human PD-1 with Chinese Hamster Ovary (CHO-K1) cells presenting human PD-L1. Following the manufacturer’s protocol, we pretreated CHO-K1 cells with different doses of PIK-93 and then cocultured them with Jurkat T cells. The results showed that PIK-93 restored the activity of Jurkat T cells in a dose-dependent manner (Fig. 2H), suggesting that PIK-93 treatment may reduce the PD-1/PD-L1 interaction, resulting in the restoration of T cell activation.

### PIK-93 promotes PD-L1 ubiquitination and proteasome degradation in lung cancer cells

**Fig. 3.**
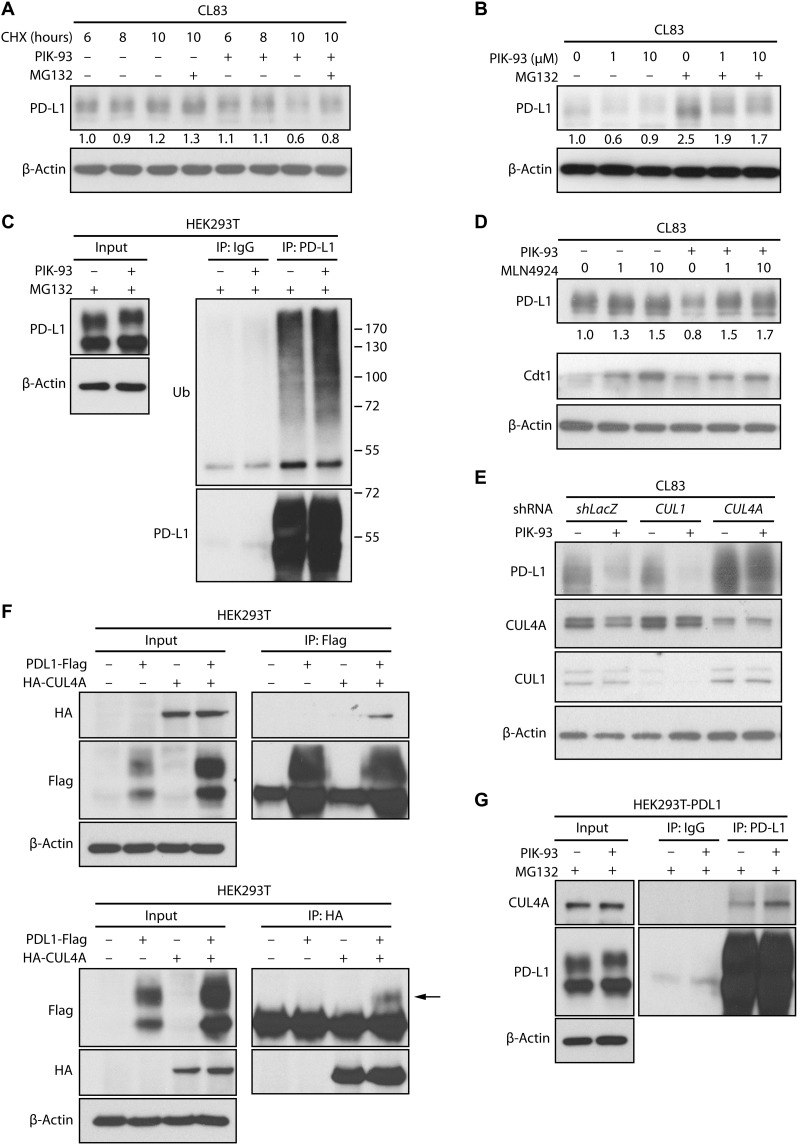
PIK-93 induces CUL4A-mediated proteasomal degradation of PD-L1. (**A**) PIK-93 reduces the half-life of endogenous PD-L1. CL83 cells were treated with cycloheximide (CHX) for the indicated time points with or without 10 μM PIK-93. Indicated proteins were examined by immunoblotting. (**B**) MG132 reverses the effects of PIK-93 on PD-L1. CL83 cells were treated with 0, 1, or 10 μM PIK-93 for 18 hours with or without 10 μM MG132 for another 6 hours. Indicated proteins were measured by immunoblotting. (**C**) PIK-93 induces ubiquitination of PD-L1. Human embryonic kidney (HEK) 293T cells were transiently transfected with PD-L1–Flag and incubated in the absence or presence of PIK-93 and MG132 (10 μM). After 6 hours, the cells were harvested, and PD-L1 was immunoprecipitated with an anti–PD-L1 antibody. Ubiquitination of PD-L1 was detected by immunoblotting. Immunoglobulin G (IgG) served as the antibody negative control for the immunoprecipitation (IP) experiment. Ub, ubiquitin. (**D**) MLN4924 restores PD-L1 protein levels. Cells were treated with DMSO or PIK-93 for 24 hours and then cotreated with different doses of MLN4924 for 6 hours. Cdt served as MLN4924-positive control. Indicated proteins were measured by immunoblotting. (**E**) CUL knockdown effect on PIK-93–induced PD-L1 polyubiquitination. CL83 cells transduced with CUL1–short hairpin RNA (shRNA), CUL4A-shRNA, or shLacZ control lentiviruses were incubated with or without PIK-93. Indicated proteins were detected by immunoblotting. (**F**) PD-L1 and CUL4A form an interaction complex. PD-L1–Flag and hemagglutinin (HA)–CUL4A were overexpressed in HEK293T cells. Reciprocal co-IP was conducted with an anti-Flag antibody (top panel) or an anti-HA antibody (bottom panel) followed by immunoblotting. (**G**) PIK-93 induces the interaction of PD-L1 and Cullin-4A. HEK293T cells were transiently transfected with PD-L1–Flag and then incubated with or without PIK-93 and MG132 (10 μM). After 6 hours of treatment, the cells were harvested, and PD-L1 was immunoprecipitated with an anti–PD-L1 antibody, followed by immunoblotting.

To determine whether the reduction in PD-L1 expression results from translational control or improved degradation, we performed a cycloheximide chase assay to inhibit protein translation in CL83 cells. As shown in Fig. 3A, the protein level of PD-L1 was reduced by PIK-93 treatment within 10 hours compared with the effect of the dimethyl sulfoxide (DMSO) control and was restored by treatment with the proteasome inhibitor MG132, indicating that PIK-93 may reduce PD-L1 protein levels via accelerated degradation. This finding was further corroborated with another experiment in which MG132 treatment led to PD-L1 protein accumulation in cells treated with different doses of PIK-93 (Fig. 3B).

We next performed an immunoprecipitation (IP) assay to pull down PD-L1 from cells with or without PIK-93 treatment and then detected polyubiquitinated PD-L1 by immunoblotting. Consistent with the previous result, treatment with PIK-93 substantially enhanced the ubiquitination of PD-L1 in human embryonic kidney (HEK) 293T cells (Fig. 3C). These data showed that PIK-93 reduces the protein level of PD-L1 via proteasome-dependent ubiquitination. To determine whether PI3K-mediated signaling could influence PD-L1 ubiquitination, we added the PI3K inhibitor LY294002 and performed PD-L1 IP to observe the levels of PD-L1–ubiquitinated species. The data indicated that there was no notable difference in PD-L1 ubiquitination between DMSO and LY294002 (lanes 1 and 3; fig. S3E). Together, our finding suggests that PD-L1 expression is suppressed by PIK-93 directly through ubiquitination, instead of being inhibited indirectly via the PI3K pathway.

### PIK-93 enhances the PD-L1–Cullin-4A interaction and PD-L1 ubiquitination

Because PD-L1 has been reported to be a substrate of cullin-3–based Ub ligase (*30*), we tested whether cullins play a role in PIK-93–induced PD-L1 degradation. We treated CL83 cells with a cullin inhibitor, MLN4924, and found that MLN4924 could restore PIK-93–decreased PD-L1 expression (Fig. 3D). To identify the cullin protein critical for PIK-93–induced PD-L1 degradation, we next assessed the effects of certain cullins on PD-L1 by small interfering RNA–mediated knockdown. We transduced CL83 cells with different short hairpin RNAs (shRNAs) against various cullins and found that CUL4A knockdown increased the PD-L1 expression (Fig. 3E). Because CUL4A is critical for the ubiquitination and degradation of intracellular targets (*31*), we tested whether CUL4A and PD-L1 interact with each other. The reciprocal IP assay with overexpressed PD-L1–Flag and hemagglutinin (HA)–CUL4A showed that PD-L1 was associated with CUL4A (Fig. 3F). Moreover, the interaction between endogenous CUL4A and PD-L1 in HEK293T-PD-L1-Flag–overexpressing cells was further enhanced by PIK-93 treatment (Fig. 3G). Together, these findings suggest that PIK-93 promotes CUL4A-mediated PD-L1 ubiquitination and degradation by increasing the interaction between CUL4A and PD-L1.

### PIK-93 decreases PD-L1 abundance on proinflammatory macrophages and enhances their antitumor effect

**Fig. 4.**
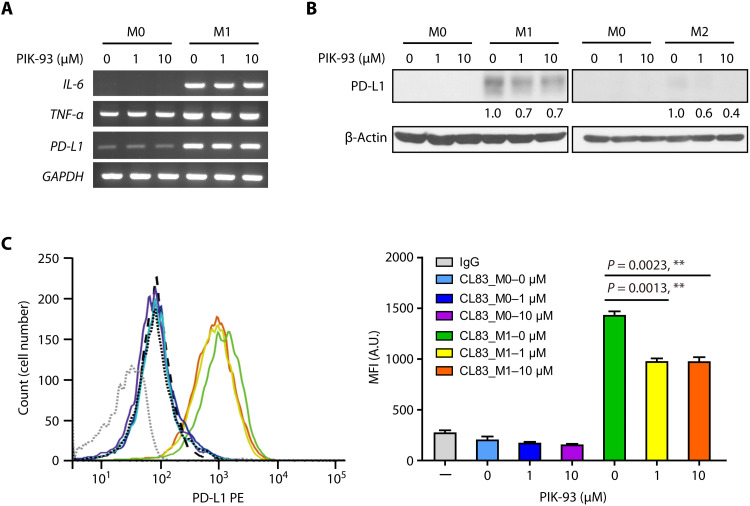
PIK-93 reduces PD-L1 abundance on M1 macrophages. (**A**) PIK-93 treatment does not affect PD-L1 RNA levels during THP-1 monocyte differentiation into M0 and M1 macrophages. After induction, the cells were treated with the indicated concentrations of PIK-93 and harvested after 24 hours. The mRNA levels of *IL-6*, *tumor necrosis factor*–α (*TNF*-α), and *PD-L1* in M0 and M1 macrophages were measured by reverse transcription polymerase chain reaction. Glyceraldehyde-3-phosphate dehydrogenase (*GAPDH*) was used as an internal control. (**B**) PIK-93 treatment reduces the PD-L1 expression most profoundly in M1 macrophages. After induction, macrophages were treated with the indicated concentrations of PIK-93 and harvested after 24 hours. (**C**) Representative flow cytometry histograms (left) and MFI (right) indicating PD-L1 abundance on M0 and M1 macrophages untreated or treated with the indicated concentrations of PIK-93 for 24 hours. The cells were harvested, and PD-L1 expression was analyzed by flow cytometry. PE, phycoerythrin. All MFI values represent the average of three independent measurements for biological independent experiments. A.U., absorbance units. The results in (C) are expressed as means ± SEM. Significant differences between indicated groups were calculated by Student’s *t* test (**P < 0.01).

**Fig. 5.**
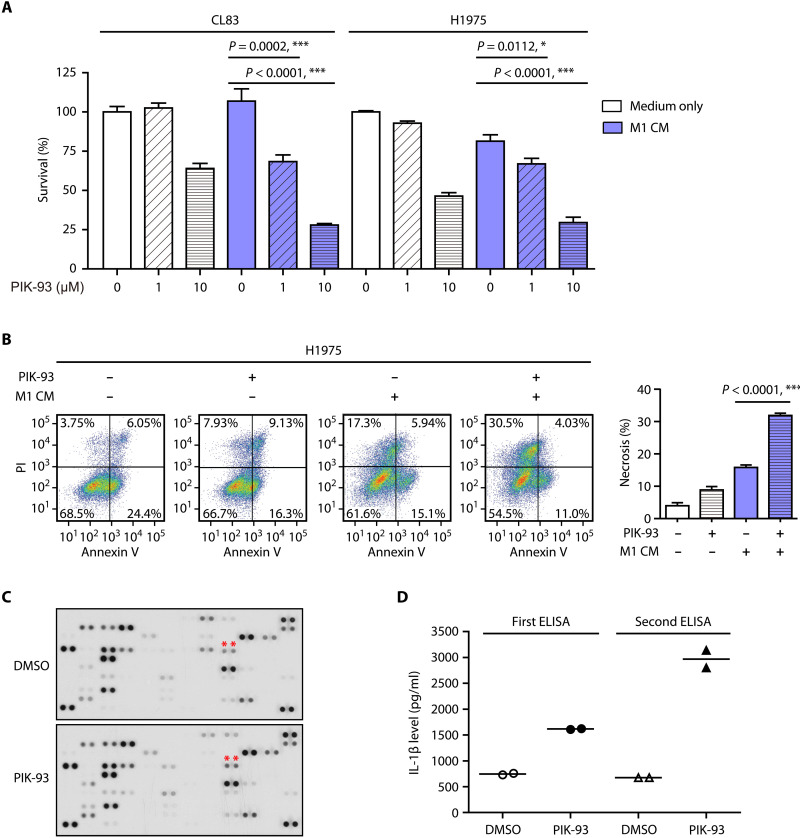
PIK-93 reduces PD-L1 abundance on M1 macrophages and enhances the anticancer function of M1 macrophages. (**A**) Modulation of lung cancer cell death by coculturing with the conditioned medium from M1 macrophages (M1 CM) pretreated with PIK-93. CL83 or H1975 cells were incubated with either medium or the M1 CM pretreated with or without 10 μM PIK-93. After 72 hours of incubation, cell viability was measured by the MTS assay. The data are shown as the means and SEM for technical and biological three independent experiments (*n* = 3). (**B**) The M1 CM pretreated with PIK-93 increases the number of necrotic H1975 cells. After incubation with either medium or the M1 CM pretreated with or without 10 μM PIK-93, H1975 cells were detached and stained with Annexin V–fluorescein isothiocyanate (FITC) and propidium iodide (PI) before fluorescence analysis was performed by flow cytometry. (**C**) The cytokine profile of the M1 CM pretreated with PIK-93. M1 macrophages were treated with or without 10 μM PIK-93 for 24 hours. The CM was harvested, and the levels of the cytokine proteins included in the Human XL Cytokine Array were measured. (**D**) The levels of IL-1β collected from M1 macrophages treated with or without 10 μM PIK-93 for 24 hours were measured by ELISA. The data are reported as the means and SEM of two independent experiments (*n* = 2) for biological independent experiments. The results in (A) and (B) are expressed as means ± SEM. Significant differences between indicated groups were calculated by Student’s *t* test (*P < 0.05 and ***P < 0.001).

TAMs are the key components that cause immunosuppression in the TME, an effect that is partially caused by PD-L1 presence on TAMs (*32*). To investigate the effect of PIK-93 on PD-L1 in TAMs, we generated macrophages from human THP-1 monocytes by incubating the THP-1 cells with phorbol 12-myristate 13-acetate (PMA). THP-1 (PMA), THP-1 [PMA + IFN-γ + lipopolysaccharide (LPS)], and THP-1 (PMA + IL-4) cells were designated M0, M1, and M2 macrophages, respectively. The RNA expression of M1 phenotype markers [*IL-6* and *tumor necrosis factor*–α (*TNF*-α)] was elevated in M1 cells compared to M0 cells, indicating that THP-1 cells had successfully differentiated into M1 cells (Fig. 4A). Notably, the RNA level of *PD-L1* was also increased in M1 cells, but it was not modulated by PIK-93 (Fig. 4A). In contrast, compared to that in M0 cells, the protein expressions of cytosolic and mPD-L1were markedly increased in M1 cells and were attenuated by PIK-93 treatment (Fig. 4, B and C), indicating that PIK-93 degrades PD-L1 in M1 macrophages. To examine whether PIK-93 promotes the tumor-killing function of M1 macrophages, the conditioned medium from M1 macrophages (M1 CM) with PIK-93 pretreatment was collected and cocultured with CL83 and H1975 cells for 72 hours. The MTS assay showed that the cytotoxic effect of the M1 CM pretreated with PIK-93 was significantly increased with the elevation of PIK-93 doses, compared to that of the M1 CM without PIK-93 pretreatment (Fig. 5A). Moreover, the M1 CM pretreated with PIK-93 significantly increased the percentage of propidium iodide (PI)–positive H1975 cells (late apoptotic and necrotic cells) compared to the M1 CM without PIK-93 pretreatment (Fig. 5B). These data demonstrate that PIK-93 enhances the tumor-killing effect of M1 macrophages by reducing PD-L1 expression on M1 macrophages.

To characterize the cytokine changes induced by PIK-93 in M1 CM that promote cytotoxicity of M1 macrophages, a human cytokine antibody array was used. The M1 CM pretreated with PIK-93 was collected, and the assay was then performed with the cytokine antibody array. M1 macrophages treated with DMSO were used as controls. The results showed that treatment with PIK-93 increased the IL-β signal intensity compared to the control (Fig. 5C). To verify the results, the M1 CM was harvested, and the level of IL-β proteins was measured by enzyme-linked immunosorbent assay (ELISA). The results showed a marked increase in IL-β after compound treatment (Fig. 5D). Proinflammatory cytokines such as IL-1β have been shown to be secreted by M1 macrophages during innate host defense and tumor killing (*33*).

To examine whether PIK-93 directly promotes the tumor-killing function of M1 macrophages or indirectly through the PI3K pathway, M1 CM pretreated with PIK-93 or the PI3K inhibitor LY294002 was collected and cocultured with CL83 and H1975 cells for 72 hours. The MTS assay showed that the cytotoxic effect of the M1 CM pretreated with LY294002 was slightly increased compared to that of the DMSO control in CL83 cells (lane 4 versus lane 6; fig. S4A), implying that the PI3K pathway may play certain minor roles in the tumor-killing function of M1 macrophages in CL83 cells. However, there was no difference in cytotoxicity between M1 CM pretreated with LY294002 and that from DMSO in H1975 cells. Western blotting was conducted to detect IL-1β production in the same M1 CM. Only PIK-93 treatment increased IL-1β production, suggesting that PIK-93 may directly regulate IL-1β production (fig. S4B). Together, these results demonstrate that PIK-93 enhances the antitumor effect of M1 macrophages on cancer cells by directly activating the IL-1β signaling pathway in M1 macrophages.

### Combined treatment with PIK-93 and anti–PD-L1 antibodies induces in vivo T cell activation, tumor growth inhibition, and increased TIL recruitment

**Fig. 6.**
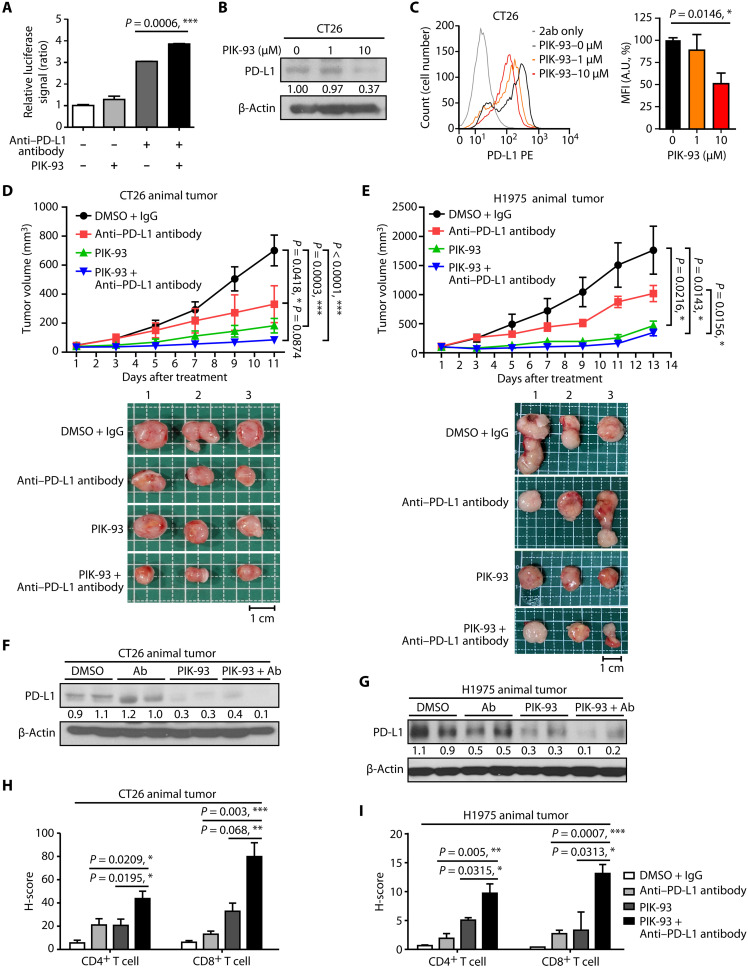
Combined treatment with PIK-93 and anti-PDL1 antibodies induces T cell activity, increases TIL recruitment, and enhances PD-L1 blockade efficacy in syngeneic mouse tumor model and human PBMC CDX mouse model in vivo. (**A**) Combined treatment with PIK-93 and anti-PDL1 antibodies induced T cell activity. CHO-K1 cells were pretreated with 0.1 μM PIK-93, 0.5 μM anti–PD-L1 antibodies, or combined treatment for 24 hours and subsequently cocultured with Jurkat T cells for another 6 hours. The relative luciferase signal indicates the activity of Jurkat T cells. (**B** and **C**) The PD-L1 level in the CT26 mouse cancer cell line is reduced after treatment with PIK-93. Cells were harvested, the cytosolic expression of PD-L1 was measured by immunoblotting (B), and the mPD-L1 level was measured by flow cytometry (C). (**D** and **E**) The combined treatment inhibits tumor growth in CT26 syngeneic BALB/c mice (*n* = 8 for each group) (D) and H1975 xenograft in humanized mice (NOD.Cg-*Prkdc^scid^ Il2rg^tm1Vst^*/Vst) (*n* = 4 for each group) (E). Top panel: The tumor curve of both models treated with DMSO + IgG control, anti–PD-L1 antibodies, PIK-93, and combination. Bottom panel: Representative pictures of the tumor mass. *n* = 3 per group. Scale bars, 1 cm. (**F** and **G**) PD-L1 in both CT26 and H1975 mouse models is degraded by PIK-93 treatment and combined treatment in vivo. Tumor samples resected from each mouse group were blotted with anti–PD-L1 antibodies. Ab, antibody. (**H** and **I**) The H-scores of CD4^+^ and CD8^+^ tumor-infiltrating leukocytes (TILs) are increased in the combined treatment. Immunohistochemistry (IHC) staining of CD4^+^ and CD8^+^ in tumor specimens after the indicated treatments. The results in (A), (C), (H), and (I) are expressed as means ± SEM for biological three independent experiments. Significant differences between indicated groups were calculated by Student’s *t* test (*P < 0.05, **P < 0.01, and ***P < 0.001).

To evaluate the immune response to cotreatment with PIK-93 and anti–PD-L1 antibodies, we first conducted a PD-1/PD-L1 blockade bioassay in which CHO-K1 cells were pretreated with PIK-93 and anti–PD-L1 antibodies separately or in combination for 24 hours and subsequently cocultured with Jurkat T cells for another 6 hours. The relative luciferase signal intensity indicated that the Jurkat T cells were more significantly activated after the combination treatment than after either PIK-93 or anti–PD-L1 antibody alone (Fig. 6A).

To assess the in vivo drug efficacy, we adopted a syngeneic mouse model (*34*) and a human PBMC CDX mouse model (*35*). First, we treated the CT26 mouse colon cancer cell line with PIK-93 and confirmed that cytosolic and mPD-L1 expressions were diminished by PIK-93 (Fig. 6, B and C). Then, we subcutaneously inoculated CT26 cells into BALB/c mice and administered the following substances intraperitoneally once every other day into mice (*n* = 8 for each group): (i) DMSO with the mouse immunoglobulin G (IgG) antibody control, (ii) anti–PD-L1 antibodies alone (6 mg/kg), (iii) PIK-93 alone (60 mg/kg), or (iv) a combination of PIK-93 and anti–PD-L1 antibodies. Figure 6D shows that treatment with PIK-93 or anti-PDL1 antibodies alone significantly inhibited tumor growth and that the combination treatment exerted an enhanced tumor-suppressing effect (*P* < 0.0001, compared with the DMSO-treated group; *P* = 0.0874, compared with the anti-PDL1 antibody–treated group). Because there is no significant difference in the combination treatment compared with the anti-PDL1 antibody–treated group in Fig 6D, we tested the expression of IL-1α, which is a tumor-suppressive cytokine (*36*, *37*), by an ELISA assay using the CT26 animal samples. The result showed that IL-1α was increased in the combination treatment with anti–PD-L1 antibodies and PIK-93 compared with anti-PDL1 antibodies only (fig. S5A). The body weights of the mice and the biochemical markers of liver and kidney functions, including aspartate transaminase, alanine transaminase, blood urea nitrogen, and creatinine, were not significantly affected, indicating that PIK-93 was well tolerated at the dose of 60 mg/kg (fig. S5, B to F). We next used subcutaneous xenografts of H1975 lung cancer cells in immunodeficient mice (NOD.Cg-*Prkdc^scid^ Il2rg^tm1Vst^*/Vst mice). After the tumor volume reached 100 mm^3^, the mice were intravenously injected with 10^7^ PBMCs. The mice were then injected intraperitoneally with (i) DMSO with the human IgG antibody control, (ii) anti–PD-L1 antibody atezolizumab only (10 mg/kg), (iii) PIK-93 only (60 mg/kg), or (iv) a combination of PIK-93 and anti–PD-L1 antibodies once every other day (*n* = 4 for each group). Figure 6E shows that treatment with only PIK-93 or the anti-PDL1 antibodies inhibited tumor growth and that the combined treatment exerted a better tumor-suppressing effect (*P* = 0.0143, compared with the DMSO-treated group; *P* = 0.0156, compared with the anti-PDL1 antibody–treated group). Similar to the syngeneic mouse model shown above, the body weights of the immunodeficient mice and levels of the biochemical markers of liver and kidney functions were not notably affected (fig. S6, A to E). The Western blot of tumor samples resected from each mouse group showed PD-L1 degradation after PIK-93 treatment and combined treatment (Fig. 6, F and G), demonstrating that PIK-93 potentially induces PD-L1 degradation in vivo.

PD-L1 expression and the recruitment of tumor-infiltrating CD4^+^ and CD8^+^ T lymphocytes were further assessed in harvested CT26- and H1975-derived tumors. Immunohistochemistry (IHC) staining of tumor specimens showed that PIK-93 treatment induced a substantial increase in the number of CD4^+^ and CD8^+^ TILs (Fig. 6, H and I), suggesting that PIK-93 may have prompted the activation and/or recruitment of CD4^+^ T helper and CD8^+^ cytotoxic T cells to the TME via PD-L1 degradation. The combination therapy of PIK-93 and anti–PD-L1 antibodies significantly increased both CD4^+^ and CD8^+^ TIL infiltration into the TME, suggesting that the combination therapy attenuates immunosuppression and enhances tumor growth inhibition in human PBMC CDX mouse models (H1975) and mouse syngeneic tumor models (CT26). To observe the number and the phenotype of TAMs in vivo, we also performed IHC staining of the H1975 animal tumor samples. The results indicated that PIK-93 treatment increased the M1 macrophage marker [inducible nitric oxide synthase (iNOS) expressions] but not M2 macrophage marker (CD163 expression) (fig. S6G), implying that more M1 macrophages were infiltrated into tumors with PIK-93 treatment. Together, these data suggest that targeting the PD-1/PD-L1 axis with PIK-93 and anti–PD-L1 antibodies further increases the recruitment of not only CD4^+^/CD8^+^ TILs but also M1 macrophages, thus enhancing the antitumor immune response to cancer immunotherapy.

**Fig. 7.**
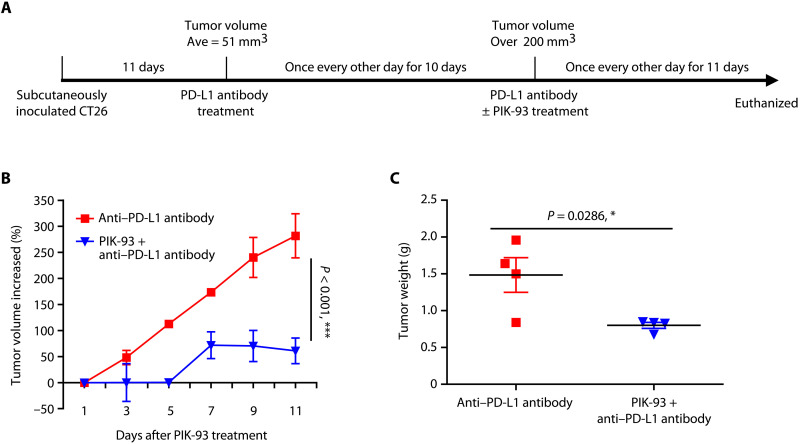
The combination of PIK-93 with anti–PD-L1 antibodies improves the antitumor response in the CT26 animal model resistant to anti–PD-L1 antibodies. (**A**) Experimental design of the CT26 animal model resistant to anti–PD-L1 antibodies and timeline of each therapy. (**B**) The percentages of tumor volume increased show that combination treatment with PIK-93 and anti-PDL1 antibodies significantly inhibited tumor growth. Tumor volume increased (%) = [(*V − V*_0_)/*V*_0_] * 100. *V*_0_ is the volume at the start of PIK-93 treatment. The percentages of tumor volume increased are shown as the means ± SD. The linear mixed model was used to examine whether changes in tumor volume over time were different between DMSO control + anti-mouse PD-L1 antibody (6 mg/kg) and the combination of PIK-93 (60 mg/kg) + anti-mouse PD-L1 antibody (6 mg/kg) groups (*n* = 4 for each group). Increased tumor volume was significantly different between two groups (***P < 0.001). (**C**) The combination treatment reduced tumor weight at the time of sacrifice (*P* = 0.0286). The results in (C) are expressed as means ± SEM. Significant differences between indicated groups were calculated by Student’s *t* test (*P < 0.05).

Last, we tested whether the combination of PIK-93 with anti–PD-L1 antibodies could improve the antitumor response in an ICI nonresponsive model (Fig. 7A). We subcutaneously inoculated CT26 cells into BALB/c mice. When the average tumor volume reached 51 mm^3^, we administered anti–PD-L1 antibodies (6 mg/kg) intraperitoneally into mice once every other day. Mice with the average tumor volume more than 200 mm^3^ were considered as not responding to the ICI therapy with anti–PD-L1 antibodies and were given: (i) DMSO control + anti-mouse PD-L1 antibody (6 mg/kg) or (ii) the combination of PIK-93 (60 mg/kg) + anti-mouse PD-L1 antibody (6 mg/kg; *n* = 4 mice for each group). The increased tumor percentage shows that combination treatment with PIK-93 and anti-PDL1 antibodies significantly inhibited tumor growth (*P* < 0.001; Fig. 7B) and reduced the tumor weight (*P* = 0.0286; Fig. 7C), compared to anti–PD-L1 antibodies alone. Thus, our results indicate that the combination of PIK-93 with anti–PD-L1 antibodies could further enhance the antitumor immune response and modulate the TME to improve immune checkpoint blockade response.

## DISCUSSION

**Fig. 8.**
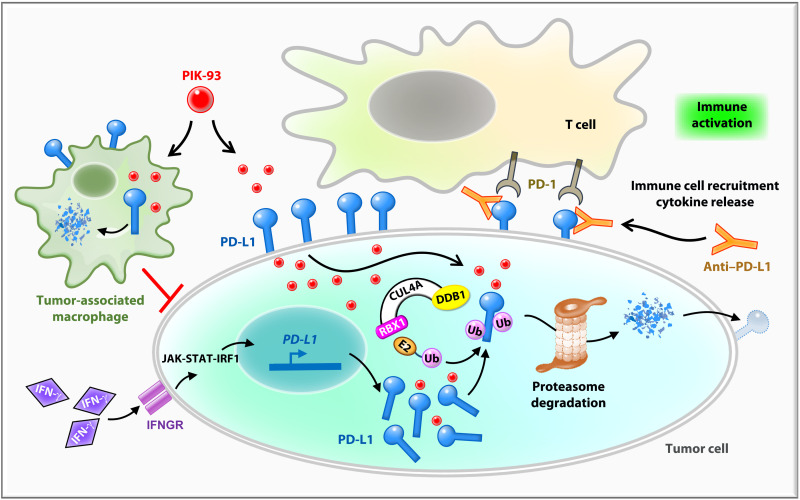
The model showing that PIK-93 combined with anti–PD-L1 antibodies blocks the PD-1/PD-L1 pathway in the TME. While PD-L1 on tumor cells suppresses T cell activation by binding to PD-1 on T cells, PD-L1 on tumor-associated macrophages (TAMs) contributes to the overall immunosuppressive microenvironment. PIK-93 treatment decreases PD-L1 proteins via E3 ligase CUL4A-facilitated proteasomal degradation. At the same time, a PIK-93–mediated PD-L1 protein reduction on tumor cells leads to increased T cell activity via PD-1/PD-L1 blockade and IFN-γ–induced PD-L1 abundance was also blocked. On the other hand, the reduction of PD-L1 on M1 macrophages by PIK-93 treatment enhances the cytotoxic activity of M1 macrophages. Last, combined treatment with PIK-93 and anti–PD-L1 antibodies increases T cell activity and immune cell recruitment to inhibit lung cancer growth. JAK-STAT-IRF1, Janus kinase–signal transducer and activator of transcription–interferon regulatory factor 1; IFNGR, interferon-gamma receptor; DDB1, DNA damage-binding protein 1; RBX1, ring-box 1.

In this study, we identified PIK-93, a PI4K inhibitor, as an effective antitumor agent that down-regulates PD-L1 protein expression in cancer cells and M1 macrophages. PIK-93 reduces PD-L1 expression in different lung cancer cell lines while inducing low cytotoxicity and restoring T cell activity. PIK-93 increases the interaction among the E3 Ub ligase, CUL4, and PD-L1, thus promoting PD-L1 proteasomal degradation. PIK-93 also decreases PD-L1 expressions on THP-1–derived M1 TAMs and reduces immunosuppression in the TME. PIK-93 combined with anti–PD-L1 antibodies suppresses tumor growth and increases the recruitment of tumor-infiltrating CD4^+^/8^+^ T cells to the TME in both mouse models. Together, PIK-93 alleviates PD-L1–mediated immunosuppression of T cells and macrophages, leading to immune activation and a favorable tumor-killing microenvironment (Fig. 8).

Previous studies have shown that the PD-L1 fraction consists of mPD-L1 (*38*), cytoplasmic PD-L1 (cPD-L1) (*39*), nuclear PD-L1 (nPD-L1) (*40*), and serum PD-L1 (sPD-L1) (*41*). mPD-L1 binds with PD-1 on the T cell surface and mediates tumor cell immune escape. Anti–PD-L1 antibodies competitively bind to mPD-L1, blocking the PD-1/PD-L1 interaction and hence activating immunity. Anti–PD-L1 antibody therapies, however, are less effective against sPD-L1 and nPD-L1 and cannot modulate cPD-L1 (*42*). In contrast, PIK-93 decreases not only cPD-L1 levels but also mPD-L1 levels (Fig. 2G) and de facto restored T cell activity (Fig. 2H). Thus, PIK-93 combined with anti–PD-L1 antibodies is an improved immunotherapy strategy to boost antitumor immunity in vitro and in vivo (Figs. 6 and 7). Previously, CUL4A has been shown to be involved in numerous cellular signaling pathways, including those involved in the cell cycle (*43*), senescence (*44*), autophagy (*45*), and apoptosis (*46*). We are the first group to validate CUL4A modulation of tumor-induced immunosuppression via the facilitation of PD-L1 degradation. Although the genomic sequences of CUL4A and CUL4B are 82% identical, they recognize different subsets of target proteins. Primarily localized in the cytoplasm, CUL4A mediates ubiquitination and degradation of intracellular targets (*31*). In contrast, CUL4B contains a nuclear localization signal and is crucial for nuclear protein degradation (*47*). Hence, CUL4A-mediated degradation of mPD-L1 and cPD-L1 explains, at least in part, the mechanism of action of PIK-93.

Numerous studies have confirmed the high expression levels of PD-L1 in TAMs; however, the function of PD-L1 in macrophages is controversial, with the initial observation suggesting that PD-L1 causes T cell anergy and inhibits proliferation (*48*). Later studies have found that PD-L1 on macrophages can limit excessive T cell activation, enhance macrophage proliferation, and increase the M2 marker expression (*49*). Tumor macrophages treated with anti–PD-L1 antibodies remodel their compartments toward a proinflammatory phenotype due to the elevation of IFN-γ (*50*). Other factors, such as the nuclear factor κB kinase inhibitor IκK-16 (*51*), pyruvate kinase M2 (PKM2) inhibitor TEPP-46 (*52*), or secreted phosphoprotein 1 (*53*), can also increase PD-L1 and M2 markers in THP-1 cells. These studies show that excessively high levels of PD-L1 in macrophages are correlated with an immunosuppressive TME, which supports tumor growth and leads to a poor survival outcome. Our data demonstrate that PIK-93 treatment reduces the PD-L1 expression level on M1 macrophages along with tumor cells, suggesting that PIK-93 may potentially exert additive effects on more than one kind of PD-L1–positive cell in the TME, thus producing strong antitumor immunity.

In summary, our study highlights an important role played by PIK-93 in CUL4-mediated degradation of PD-L1 on tumor cells and macrophages, resulting in restored antitumor immune activity. Our findings suggest that combining the small molecule PIK-93 with monoclonal antibody–based ICIs can promote a favorable TME that enables enhanced efficacy of PD-1/PD-L1–mediated immunotherapy in lung cancer.

## MATERIALS AND METHODS

### Cell lines and culture conditions

The human lung adenocarcinoma cell CL83 with wild-type epidermal growth factor receptor was clinically isolated and cultured in our laboratory beforehand (*54*). The NCI-H1975, H522, HOP92, A549, and HEK293T human cancer cell lines were purchased from the American Type Culture Collection (Manassas, VA). The PC9 cell line was a gift from C. H. Yang (Graduate Institute of Oncology, Cancer Research Center, National Taiwan University). THP-1 monocytes were differentiated into macrophages by incubation with 150 nM PMA (Sigma-Aldrich, P8139) for 24 hours. Macrophages were polarized into M1 macrophages by incubation with IFN-γ (20 ng/ml; R&D Systems, catalog no. 285-IF) and LPS (10 pg/ml; Sigma-Aldrich, catalog no. 8630). Macrophage M2 polarization was performed by incubation with IL-4 (20 ng/ml; R&D Systems, catalog no. 204-IL). Cells were grown in RPMI 1640 or Dulbecco’s modified Eagle’s medium with 10% fetal bovine serum (v/v) and penicillin (100 U/ml) or streptomycin (100 μg/ml) in a humidified atmosphere at 37°C with 5% CO_2_ to 95% air.

### IL-2 secretion by activated T cells

H1975 cells were pretreated with the drug PIK-93 for 24 hours. These cells were cocultured with Jurkat T cells for an additional 24 hours with the Cell Stimulation Cocktail (eBioscience/Thermo Fisher Scientific, Waltham, MA). Anti–PD-L1 antibodies (10 ng/ml) or the mouse IgG isotype control was added to the coculture medium. Supernatants were collected after 48 hours, and IL-2 expression level was measured with an IL-2 human ELISA kit (Thermo Fisher Scientific, MA, USA).

### Immunofluorescence staining

The cells were fixed at room temperature in 3.7% cold paraformaldehyde in phosphate-buffered saline (PBS) for 10 min and permeabilized at room temperature with PBS containing 0.1% Triton X-100 for 10 min. The cells were blocked with PBS containing 3% bovine serum albumin (BSA). The cells were then mounted with ProLong Gold antifade reagent containing 4′,6-diamidino-2-phenylindole (DAPI; Molecular Probes, Eugene, Oregon) and examined and photographed with a Zeiss LSM 700 confocal microscope (Zeiss, Urbana, IL).

### High-throughput screening

The *IRES* fragment was amplified by polymerase chain reaction amplification using the forward primer: cgcggatccagatctatgatgtgttaagatccgcccctc and the reverse primer: cgcggatcccatggttgtggccatattatc and cloned into pcDNA3-*mRuby2*, which was obtained from B.-T. Huang. The IRES-mRuby2 was then subcloned into pEGFP-C1 to generate pEGFP-IRES-mRuby. To construct p*PD-L1-EGFP-IRES-mRuby*, the *PD-L1* fragment from hB7-H1 VersaClone cDNA (R&D Systems) was clone into pEGFP-IRES-mRuby.

Drug screening was performed using a compound library from P.C. Yang’s laboratory in 96-well plates and an HTS system manufactured in Academia Sinica (Taipei, Taiwan). For preliminary screening, H1975-PD-L1-EGFP cells were dispensed into the wells of a 96-well plate at a concentration of 4000 cells per well. The cells were incubated for 1 day in an environmentally controlled incubator (37°C, 5% CO_2_, and 80% humidity), and fluorescence was monitored using an MD-ImageXpress Micro Imaging XL System after compound library treatment for 1 day. The IC_50_ values were calculated on the basis of the EGFP/RFP ratio intensity. Compounds reducing a >25% in fluorescence were selected for further investigation. Compounds at 5 μM with <50% survival and without dose response were excluded.

### IP and immunoblotting

In brief, H1975 or CL83 cells were seeded into 10-cm plate overnight and then treated with PIK-93 or DMSO as control group. After 24 hours, the cells were harvested with a scraper, washed three times with PBS, and lysed on ice for 30 min in ice-cold IP buffer [20 mM tris, 150 mM NaCl, 100 μM Na_3_VO_4_, 50 mM NaF, 30 mM sodium pyrophosphate, 0.5% NP-40 (Sigma-Aldrich), and protease inhibitor cocktail tablet (Roche Diagnostics, Basel, Switzerland)]. The cell lysates were passed 10 times through a 21-gauge needle and processed by centrifugation at 12,000 revolutions per minute (rpm) for 30 min at 4°C. The supernatants protein was determined with Bicinchoninic Acid Assay Protein Assay Reagent (Pierce, Rockford, IL, USA) using BSA as the standard. After adding Protein A–Sepharose (GE HealthCare) beads for 1 hour, the lysates were washed with ice-cold PBS five times. Equivalent amounts of the proteins were resolved by 8 to 10% SDS–polyacrylamide gel electrophoresis and transferred to a polyvinylidene difluoride membrane (Millipore, Billerica, MA). The membranes were blocked with 5% nonfat dried milk in TBST buffer [10 mM tris (pH 7.5), 100 mM NaCl, and 0.05% Tween 20] for 1 hour and then were incubated with respective primary antibodies in TBST overnight at 4°C. The primary antibody of PD-L1 (cs-13684), TIM-3(cs-45208), and p-AKT (cs-4060) were purchased from Cell Signaling Technology (MA, USA); GFP (sc-9996), Ub (sc-8017), glyceraldehyde-3-phosphate dehydrogenase (GAPDH; sc-25778), CUL1 (sc-11384), and Cdt-1(sc-28262) were purchased from Santa Cruz Biotechnology Inc. (CA, USA); Flag (F7425) and β-actin (A2066) were purchased from Sigma-Aldrich (MO, USA); CERCAM-1 (GTX123293), CTLA-4 (GTX32542), HA (GTX115044), and IL-1β (GTX130021) were purchased from GeneTex (CA, USA); CD86 (ab53004), adenosine triphosphatase (ATPase; ab76020), and CUL4A (ab72548) were purchased from Abcam (England, UK); CD68 (NB100-683) was purchased from Novus Biologicals (CO, USA); CD163 (MA5-11458) was purchased from Thermo Fisher Scientific (MA, USA); and iNOS (NB300-605) was purchased from Novus Biologicals (CO, USA). On the next day, the membrane was incubated with appropriate horseradish peroxidase–conjugated secondary antibodies in TBST for 1 hour. The signal of the specific immunoreactive bands was detected with a enhanced chemiluminescence (ECL) kit following the producer’s protocol (PerkinElmer Inc., USA), quantified by densitometry and normalized against β-actin as the control with ImageJ software.

### Cell viability assay

Briefly, 5 × 10^3^ cells per well were seeded into 96-well plates in the medium before drug treatment for overnight. Then, different concentrations of PIK-93 were treated to cells for another 72 hours in the medium at 37°C. Then, 20 μl of MTS stock solution was mixed into the 100 μl of medium in each well with freshly prepared. After 1 hour of incubation, optical density at 490 nm was detected with a spectrophotometer (Molecular Devices). The IC_50_ value was defined as the drug inhibitory concentration producing a 50% decrease after drug exposure for 72 hours.

### Flow cytometry

For PD-L1 labeling, the cells were cultured for 24 hours, treated with PIK-93 for 24 hours and labeled with anti–PD-L1 antibodies. Cells were fixed and permeabilized with a Cytofix/Cytoperm Buffer with 5-bromo-2’-deoxyuridine (BrdU) Staining (BD Bioscience, San Jose, CA) and treated with anti–PD-L1 antibodies. The samples were analyzed in a BD FACSCalibur flow cytometer (Becton Dickinson, Sweden) for histogram analysis.

### Isolation of plasma membrane proteins

Cell membrane proteins were isolated from whole cells using a Plasma Membrane Protein Extraction Kit (BioVision, Mountain View, California) according to the manufacturer’s instructions as previously described (*55*).

### T cell activity assay

T cell activity was measured using the PD-1/PD-L1 Blockade Bioassay Kit (Promega, Madison, WI, USA) according to the manufacturer’s instructions.

### Lentiviral shRNA induction

HEK293T were cotransfection with the lentiviral vector pLKO.1-sh*CUL1* or pLKO.1-sh*CUL4A/B* from the National RNAi Core Facility (Academia Sinica, Taiwan) and two helper plasmids (pCMV△R8.91 and pMD.G) using Lipofectamine 2000 (Invitrogen) to produced lentiviruses. The supernatant with virus were collected 24, 48, or 72 hours after transfection, centrifuged, and filtered through 0.45-mm pore-sized filters. The cells were infected with the indicated lentivirus in medium containing polybrene (8 μg/ml). Twenty-four hours after infection, the cells were treated with fresh medium for 48 hours and used for other experiments.

### Flow cytometry analysis with annexin V/PI staining

H1975 cells were seeded in 6-cm plates 1 day before incubation with CM from macrophage cultures. After incubation, tumor cells were harvested with trypsin/EDTA and stained using fluorescein isothiocyanate (FITC) Annexin V Apoptosis Detection Kit I (BD Pharmingen, no. 556547). The results were analyzed by flow cytometry (BD FACSCalibur, BD).

### Cytokine assay

THP-1 cells were seeded in 24-well plates and treated with PMA (300 ng/ml), IFN-γ (20 ng/ml), and LPS (10 pg/ml). After 24 hours, the cells were treated with DMSO or 10 μM PIK-93 for another 24 hours in complete culture medium at 37°C. The CM was harvested and centrifuged for 5 min at 1700 rpm. The supernatants were transferred to clean microcentrifuge tubes, and equivalent amounts of control and treatment CM were incubated with the human cytokine proteome profiler array (R&D Systems) according to the manufacturer’s protocol. The membranes were then scanned, and the density was measured.

### Mouse syngeneic tumor models and human PBMC CDX mouse models

A total of 2 × 10^5^ stable CT26 cells were subcutaneously injected into the flanks of male BALB/c mice. Drug treatment was initiated on day 16 until the average tumor volume reached 40 mm^3^. Mice were randomly separated into four groups and then injected intraperitoneally with DMSO control + control rat IgG2b antibody (LTF-2, Bio X cell), DMSO control + anti-mouse PD-L1 antibody (6 mg/kg; 10F.9G2, Bio X cell), PIK-93 (60 mg/kg) + control rat IgG2b antibody, or the combination of PIK-93 (60 mg/kg) + anti-mouse PD-L1 antibody (6 mg/kg) once every other day for 2 weeks. We also subcutaneously injected H1975 lung cancer cells in immunodeficient mice (NOD.*Cg-Prkdc^scid^ Il2rg^tm1Vst^*/Vst mice) for xenograft development. After the tumor volume reached 100 mm^3^, the mice were intravenously injected with 10^7^ PBMCs. On the same day, the mice were injected intraperitoneally with DMSO control + human IgG1 antibody (HG1K, Sino Biological), DMSO control + anti–PD-L1 antibody (10 mg/kg; atezolizumab, MedChemExpress), PIK-93 (60 mg/kg) + human IgG1 antibody, or the combination of PIK-93 (60 mg/kg) + anti–PD-L1 antibody (10 mg/kg) once every other day for 2 weeks.

To simulate the TME which is not responding to ICI therapy, CT26 cells were subcutaneously injected into BALB/c mice. When the average tumor volume reached 51 mm^3^, anti–PD-L1 antibodies (6 mg/kg) were administered intraperitoneally into mice once every other day. A total of five administrations were given to mice. If the average tumor volume grew more than 200 mm^3^, then mice were considered as nonresponsive to ICI therapy with anti–PD-L1 antibodies. Then, mice were randomly separated into two groups: DMSO control + anti-mouse PD-L1 antibody (6 mg/kg) or the combination of PIK-93 (60 mg/kg) + anti-mouse PD-L1 antibody (6 mg/kg; *n* = 4 for each group). A total of six treatments were performed. During the treatment, the tumor sizes and body weights were measured every other day, with the tumor size calculated by *V* = 0.5 × (*length*) × (*width*)^2^. Animal care was conducted following the guidelines of Academia Sinica (Nankang, Taipei, Taiwan). The protocol was approved by the Committee on the Ethics of Animal Experiments of Academia Sinica (Nankang, Taipei, Taiwan, Academia Sinica Institutional Animal Care and Use Committee: 13-11-593).

### Statistical analysis

The data are shown as the means ± SEM. The significance of differences was determined by Student’s *t* test or Pearson’s chi-square test. *P* values of 0.05 were considered to be statistically significant. The percentages of tumor volume increase are shown as the means ± SD. The linear mixed model was used to examine whether changes in tumor volume over time were different between DMSO control + anti-mouse PD-L1 antibody (6 mg/kg) and the combination of PIK-93 (60 mg/kg) + anti-mouse PD-L1 antibody (6 mg/kg). *P* values of 0.05 were considered to be statistically significant.

## Notes

View/request a protocol for this paper from *Bio-protocol*.
