# Supplementary material for: Small-molecule PIK-93 modulates the tumor microenvironment to improve immune checkpoint blockade response
Source: Sci Adv. 2023 Apr 7;9(14):eade9944. doi: 10.1126/sciadv.ade9944 (PMC10081850; doi:10.1126/sciadv.ade9944)
Supplement: Supplementary file 1 — Figs. S1 to S6 Legend for table S1 [file sciadv.ade9944_sm.pdf]

Science Advances
AAAS

Supplementary Materials for

Small-molecule PIK-93 modulates the tumor microenvironment to improve immune checkpoint blockade response

Chia-Yi Lin *et al.*

Corresponding author: Pan-Chyr Yang, pcyang@ntu.edu.tw

Sci. Adv. **9**, eade9944 (2023)
DOI: 10.1126/sciadv.ade9944

The PDF file includes:

Figs. S1 to S6
Legend for table S1

Other Supplementary Material for this manuscript includes the following:

Table S1

Fig. S1

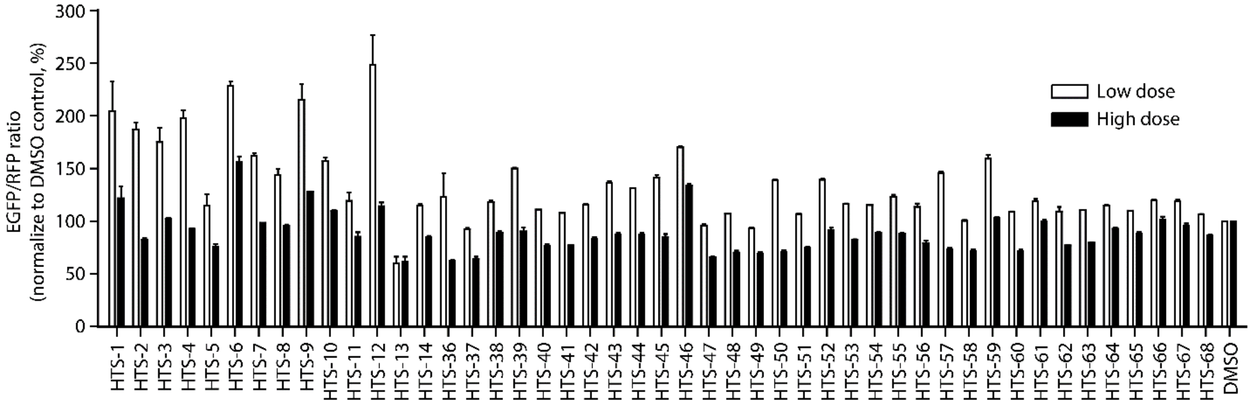

Fig. S1. HTS screening result of H1975-PD-L1-EGFP cells. H1975-PD-L1-EGFP cells were treated with compound library for 2 doses and fluorescence intensity of EGFP and RFP were analyzed with a high-content imaging-based screening system. Quantification of the relative levels of EGFP/RFP was normalized to the levels of DMSO.

Fig. S2

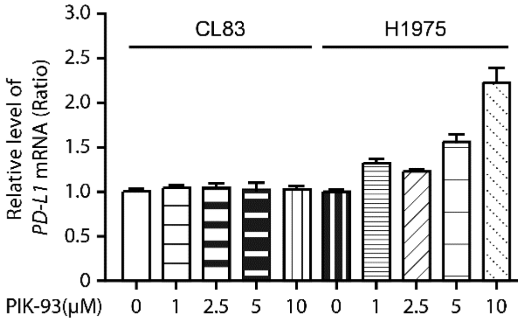

Fig. S2. *PD-L1* RNA levels after treatment with 0, 1, 2.5, 5 and 10 μM PIK-93 in CL83 and H1975 cells. PIK-93-treated cells were harvested 24 h after treatment, and *PD-L1* RNA levels were analyzed with RT–PCR. Quantification of the relative levels of *PD-L1* was normalized to the levels of *GAPDH*.

Fig. S3

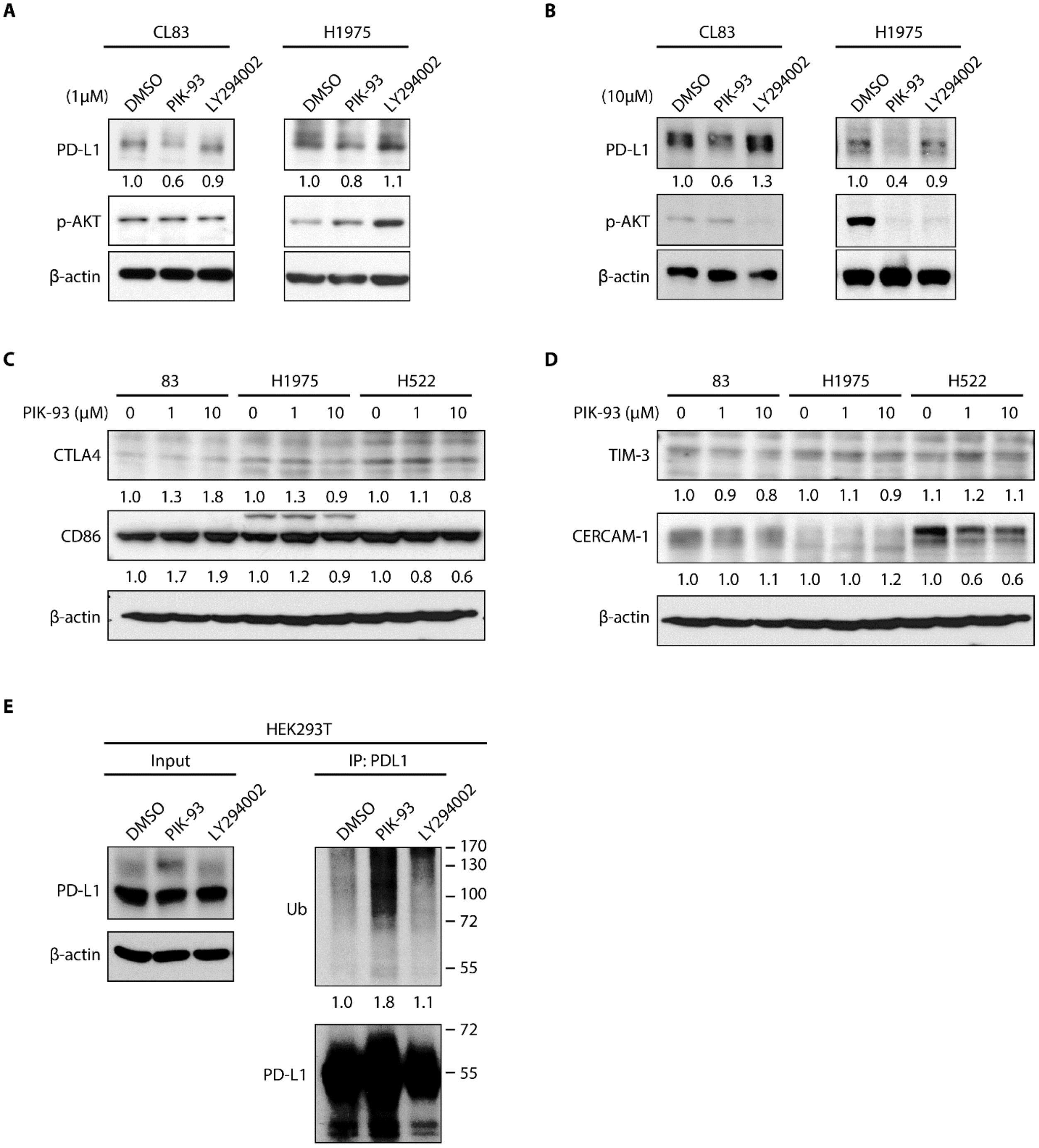

Fig. S3. PIK-93, but not LY294002, regulates PD-L1 degradation. (A) CL83 and H1975 cells were harvested at 24 h after treatment with 1 μM PIK-93 or LY294002. The levels of PD-L1 were analyzed by immunoblotting. (B) CL83 and H1975 cells were treated with 10 μM PIK-93 or LY294002 for 24 h and then harvested. The PD-L1 level was determined by immunoblotting. (C) PIK-93 treatment induces ubiquitination of PD-L1. HEK293T cells were transiently transfected with PD-L1-Flag and then incubated in the presence of PIK-93 or LY294002 (10

μM). After 6 h MG132 treatment, the cells were harvested, and PD-L1 was immunoprecipitated with an anti-PD-L1-specific antibody. Ubiquitination of PD-L1 was detected by immunoblotting. (D) CTLA4 and CD86 expression in different lung cancer cell lines with PIK-93 treatment. Cells were treated with the indicated doses of PIK-93 for 24 h and then harvested. The CTLA4 and CD86 levels were determined by immunoblotting. (E)TIM-3 and CERCAM-1 expression in different lung cancer cell lines with PIK-93 treatment. Cells were treated with the indicated doses of PIK-93 for 24 h and then harvested. The TIM-3 and CERCAM-1 levels were determined by immunoblotting.

Fig. S4

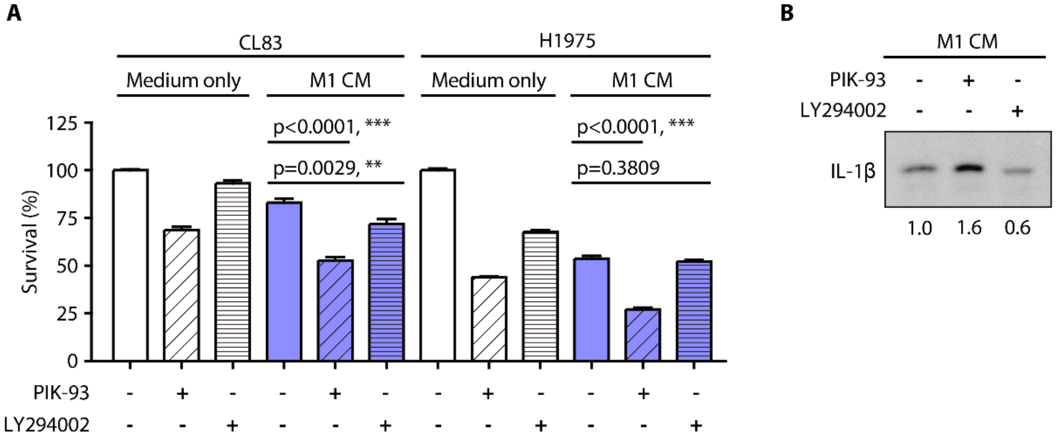

Fig. S4. PIK-93 enhances the antitumor effect of M1 macrophages on cancer cells by directly activating the IL-1β signaling pathway in M1 macrophages. (A) CL83 or H1975 cells were incubated with either medium or the M1 CM pretreated with DMSO, PIK-93, or LY294002. After 72 h of incubation, cell viability was measured by the MTS assay. (B) The levels of IL-1β collected from M1 macrophages CM pre-treated with DMSO, PIK-93, or LY294002 for 24 h were measured by immunoblotting.

Fig. S5

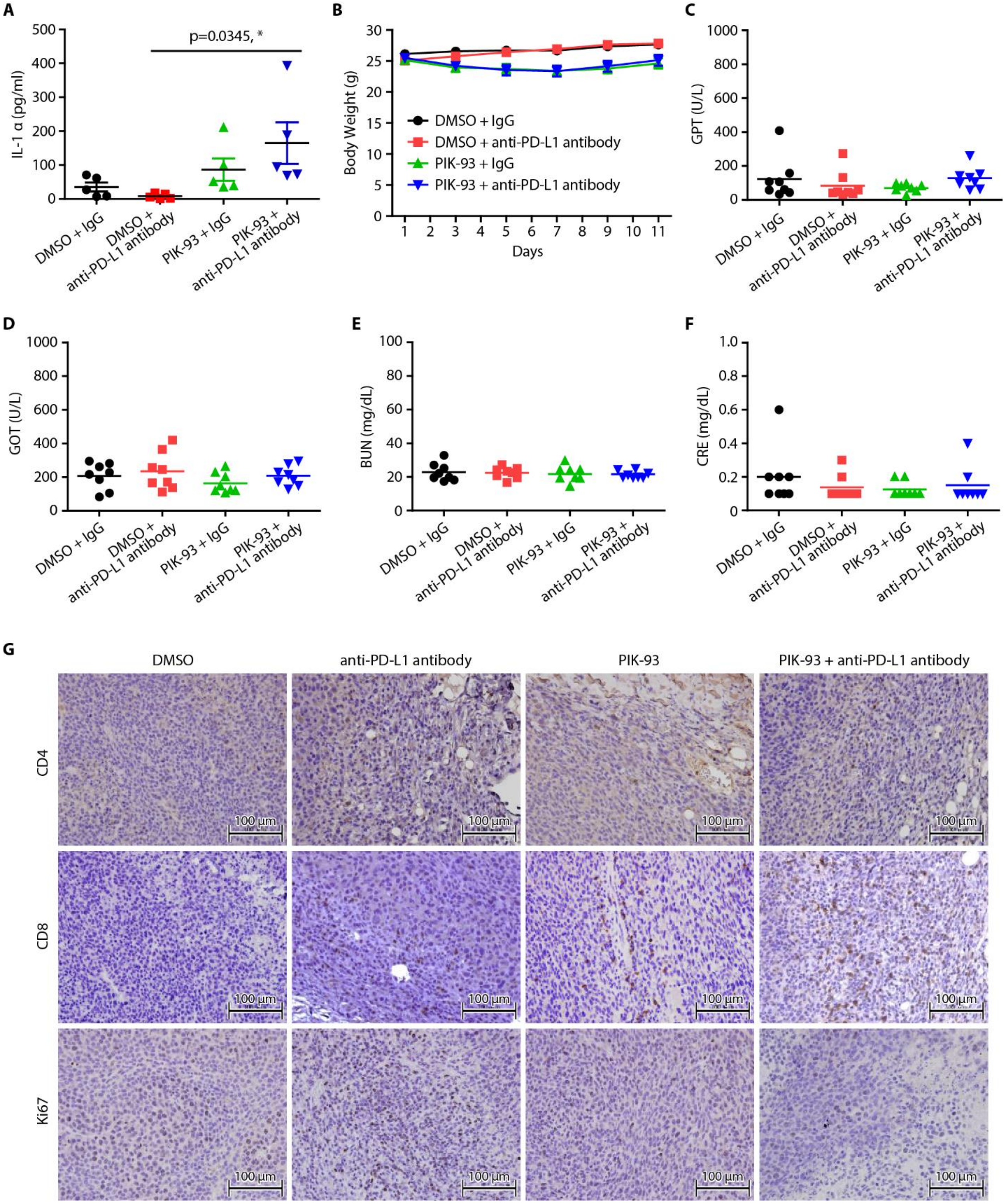

Fig. S5. CT26 syngeneic tumor model of PIK-93 combined with anti-PD-L1 antibodies. BALB/c mice that bore subcutaneous CT26 tumors were treated intraperitoneally with DMSO+ the mouse IgG antibody control, 6 mg/kg anti-PD-L1 antibodies only, 60 mg/kg PIK-93 only, or

the combination of PIK-93 and anti-PD-L1 antibodies once every other day (n = 8 for each group). **(A)** ELISA assay of IL-1α expression in the CT26 animal sample. IL-1α was increased by the combination treatment with anti-PD-L1 antibodies and PIK-93, compared with anti-PD-L1 antibodies alone (*p*=0.0345)**.** No significant differences were found between the drug-treated and DMSO-treated groups in terms of the body weight **(B)**, aspartate transaminase (GPT) **(C)**, alanine transaminase (GOT) **(D)**, urea nitrogen (BUN) **(E)** or creatinine (CRE) **(F)**. **(G)** Immunohistochemistry (IHC) staining of $CD4^+$, $CD8^+$, and $Ki67^+$ in tumor specimens with the indicated treatments. $Ki67^+$ indicates tumor proliferation. Scale = 100 μm.

Fig. S6

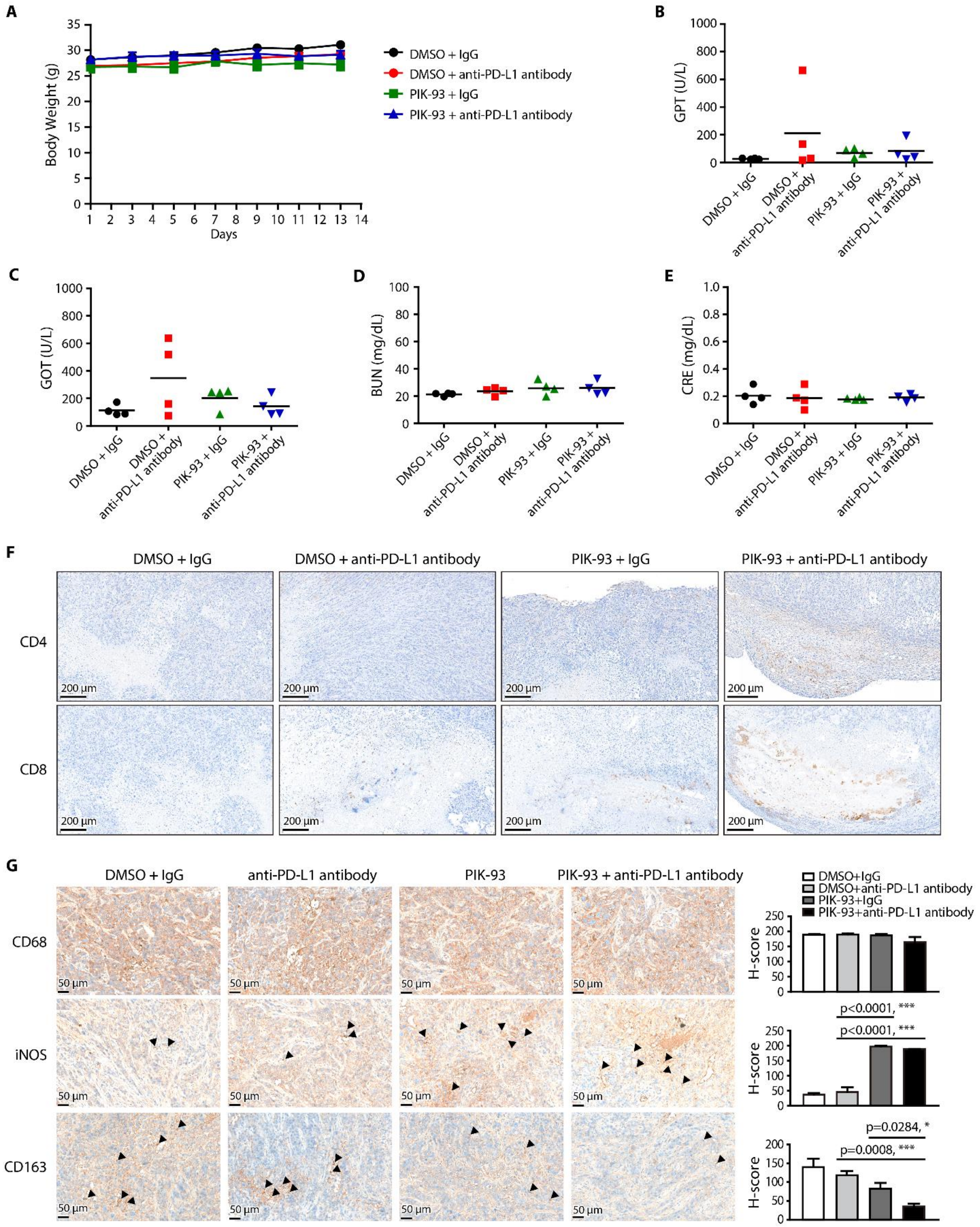
A
Body Weight (g)
Days
DMSO + IgG
DMSO + anti-PD-L1 antibody
PIK-93 + IgG
PIK-93 + anti-PD-L1 antibody
B
GPT (U/L)
C
GOT (U/L)
D
BUN (mg/dL)
E
CRE (mg/dL)
F
CD4
CD8
200 µm
G
DMSO + IgG
anti-PD-L1 antibody
PIK-93
PIK-93 + anti-PD-L1 antibody
CD68
iNOS
CD163
50 µm
DMSO+IgG
DMSO+anti-PD-L1 antibody
PIK-93+IgG
PIK-93+anti-PD-L1 antibody
H-score
p<0.0001, ***
p<0.0001, ***
p=0.0284, *
p=0.0008, ***

Fig. S6. Human peripheral blood mononuclear cell (PBMC) cell line-derived xenograft (CDX) mouse model treated with PIK-93 combined with anti-PD-L1 antibodies. H1975 lung cancer cells were subcutaneously injected into immunodeficient mice (NOD.Cg-*Prkdc*^{scid} *Il2rg*^{tm1Vst}/Vst mice). After the tumor volume reached 100 mm^3, the mice were intravenously injected with 10^7 PBMCs. The mice were then injected intraperitoneally with DMSO + the human IgG antibody control; DMSO + 10 mg/kg atezolizumab, an anti-PD-L1 antibody; 60 mg/kg PIK-93 + human IgG antibody control; or the combination of PIK-93 and atezolizumab once every other day (n = 4 for each group). No significant differences were found between the drug-treated and DMSO-treated groups body weight **(A)**, aspartate transaminase (GPT) **(B)**, alanine transaminase (GOT) **(C)**, urea nitrogen (BUN) **(D)** or creatinine (CRE) **(E)**. **(F)** Immunohistochemistry (IHC) staining of CD4$^+$ and CD8+ in tumor specimens with the indicated treatments. Scale = 200 μm. **(G)** Immunohistochemistry (IHC) staining of macrophage marker (anti-CD68), M1 macrophage (anti-iNOS) and M2 macrophage (anti-CD163) in tumor specimens with the indicated treatments. Scale = 50 μm.

Additional Supplementary materials

Supplementary tables: Table S1. Compound library list for HTS screening.
